# Spontaneous functional changes in specific cerebral regions in patients with hypertensive retinopathy: a resting-state functional magnetic resonance imaging study

**DOI:** 10.18632/aging.202999

**Published:** 2021-05-10

**Authors:** Min-Jie Chen, Shi-Nan Wu, Hui-Ye Shu, Qian-Min Ge, Yi-Cong Pan, Li-Juan Zhang, Rong-Bin Liang, Qiu-Yu Li, Wan Zhang, Yi Shao

**Affiliations:** 1 Department of Ophthalmology, The First Affiliated Hospital of Nanchang University, Jiangxi Centre of Natural Ocular Disease Clinical Research Center, Nanchang 330006, Jiangxi, People’s Republic of China; 2 Department of Cardiovascularology, The First Affiliated Hospital of Nanchang University, Nanchang 330006, Jiangxi, People’s Republic of China

**Keywords:** hypertensive retinopathy, neuroimaging, functional MRI, voxel-wise degree centrality, spontaneous cerebral activity

## Abstract

This study investigated functional alterations in the cerebral network of patients with hypertensive retinopathy (HR) by resting-state functional magnetic resonance imaging (rs-fMRI) and degree centrality (DC) methods. 31 patients with HR along with 31 healthy controls (HC) closely matched in gender and age were enrolled for the research. All participants were examined by rs-fMRI, and the DC method was applied to evaluate alterations in spontaneous cerebral activity between the 2 groups. We used the independent samples *t* test to evaluate demographic and general information differences between HR patients and HCs. The 2-sample *t* test was used to compare the DC values of different cerebral regions between the 2 groups. The accuracy of differential diagnostic HR was analyzed by receiver operating characteristic (ROC) curve method for rs-fMRI DC values changes. Pearson’s correlation coefficient was applied to determine the correlation between differences in DC in specific cerebral areas and clinical manifestation. Results showed that DC values were higher in the left cerebellum posterior lobe (LCPL), left medial occipital gyrus (LMOG), and bilateral precuneus (BP) of HR patients compared to HCs. Mean DC values were lower in the right medial frontal gyrus/bilateral anterior cingulate cortex of HR patients. Anxiety and depression scores were positively correlated with DC values of LMOG and LCPL, respectively. Bilateral best-corrected visual acuity in HR patients was negatively correlated with the DC value of BP. Hence, changes in DC in specific cerebral areas of patients with HR reflect functional alterations that provide insight into the pathophysiologic mechanisms of HR.

## INTRODUCTION

**Figure 1:**
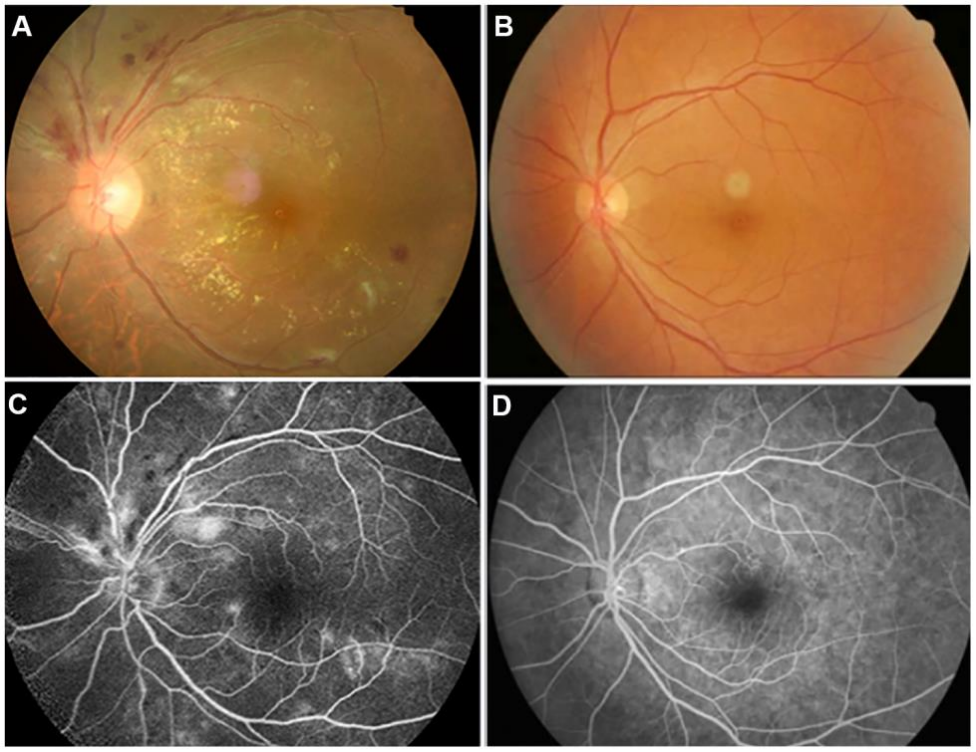
**Examples of retinal fundus photography (above) and fluorescence fundus angiography (below) in the HR patients and HC group.** Notes: (**A**) shows the left retinal fundus photos of patients with hypertensive retinopathy and (**B**) shows the left retinal fundus photos of normal people. (**C**) shows left fluorescence fundus angiography in patients with hypertensive retinopathy, and (**D**) shows corresponding fluorescence fundus angiography in normal subjects. (**A**, **C**) Correspond to the same person, and (**B**, **D**) correspond to another person.

Hypertensive retinopathy (HR) is a systemic manifestation of hypertension (Figure 1). The fundus of the eye is the only part of the body where blood vessels and any changes thereof can be directly observed, which can reveal vascular diseases in other areas [1]. Hypertension can damage the retina, choroid, and optic nerve circulation, thereby affecting the anatomic and physiologic functions of the eye including vision, [2, 3] and causing secondary morbidities such as ischemic optic neuropathy and retinal vascular occlusion that may lead to loss of vision [1]. HR is detected in 2%–14% of nondiabetic people aged ≥40 years [4]. Common signs of retinopathy in patients with hypertension include microaneurysms, cotton spots, hard exudation, and bleeding, among others. HR can be divided into vasoconstriction, hardening, and exudation stages [5]. Up to now, the main treatment modality for HR patients has been systemic antihypertensive therapy based on the severity of the disease [6].

The retina develops as part of the brain, [7] which is one of the most severely affected areas in people with hypertension [8]. The retinal artery and the cerebral microcirculation share common embryologic, anatomic, and physiologic characteristics [9]. HR patients are at risk of death from cardiovascular and cerebrovascular diseases [10]. The risk of stroke is nearly 3 times higher in patients with moderate or severe retinopathy as compared to healthy subjects. Retinal signs of HR and retinopathy are related to the slow progression of brain white matter and cerebral microvascular disease. Changes in white matter are visible as hyperintense signals by magnetic resonance imaging (MRI) [11]. Subtle changes in retinal blood vessels have been linked to subclinical cerebrovascular diseases such as stroke diagnosed by MRI and symptomatic or asymptomatic lacunar infarction in healthy individuals [12–14]. Thus, HR is not only associated with abnormalities in blood vessels and retina but also involves alterations in cerebral activity, although this has not been extensively studied.

Resting-state functional (rs-fMRI) is a noninvasive imaging technique that reveals large fluctuations in spontaneous low-frequency (<0.1 Hz) signals in functionally related cerebral areas, which is widely utilized for cerebral functional connectivity (FC) studies. Rs-fMRI detects spontaneous neuronal activity in the resting state of human brain, [15, 16] and has the advantages of simplicity and ease of application, which is useful when examining patients with reduced coordination and cerebral diseases such as Alzheimer disease, [17] multiple sclerosis, [18] and stroke. Brain MRI has revealed subclinical brain damage in asymptomatic hypertensive patients, [19] while a rs-fMRI study showed that changes in cerebral structure including gray matter abnormalities were detectable in hypertensive patients [20].

Recently, network analysis based on graph theory has been applied to investigations of brain FC. Degree centrality (DC) is a functional connection algorithm based on graph theory that is a useful adjunct to MRI [21, 22]. DC considers each voxel as a node and calculates the number of functional connections between each node and other nodes, which indirectly reflects the status of a specific brain area FC. Up to now, DC has been used to analyze neural networks in patients with type 2 diabetes and severe obstructive sleep apnea as well as ophthalmic diseases including angle-closure glaucoma, high myopia, advanced monocular blindness, open eyeball injury, and comitant exotropia strabismus [23–27] (Table 1). At present, the diagnosis of HR is mainly limited to fundus examination, and there is still a lack of relevant research on the effect of HR-induced cerebral functional activities. Severe HR patients may experience headache and significant loss of vision [28]. As DC is an important method to study the changes of cerebral functional activity, it has the advantages of non-invasiveness and convenience, and it is one of the important technologies to apply DC method to study the changes of cerebral functional activity in HR patients. However, it has not been previously applied to investigations of HR. Moreover, clinical data for cerebral activity in HR are scarce. In order to solve the above problems, we studied the changes of cerebral FC in patients with HR by rs-fMRI and DC analysis methods.

**Table 1:** DC method applied in ophthalmology-related diseases.

**Authors**	**Year**	**Diseases**
Cai et al [23]	2015	Primary angle-closure glaucoma before and after surgery
Huang et al [24]	2019	Late monocular blindness
Tan et al [25]	2018	Adult comitant exotropia strabismus
Wang et al [26]	2017	Acute unilateral open globe injury
Hu et al [27]	2018	High myopia

Abbreviation: DC, degree centrality.

## MATERIALS AND METHODS

### Participants

Our study was approved by the Medical Ethics Committee of the First Affiliated Hospital of Nanchang University, and all subjects signed the informed consent. In addition, all the research process was conducted in accordance with the Declaration of Helsinki.

A total of 31 HR patients (16 males and 15 females) were recruited at the ophthalmology department of the hospital from August 2018 to October 2019. The criteria for diagnosing HR were as follows: 1) fundus examination performed based on a systematic diagnosis of stage 2 or above hypertension (systolic blood pressure ≥ 140mmHg or diastolic blood pressure ≥ 90mmHg); [29] and 2) a record of microaneurysms, retinal hemorrhage, cotton thread spots, exudate, arteriovenous crossing, arteriolar stenosis, and papilledema, which were used to classify retinopathy.

Retinopathy was graded according to the Keith–Wagener classification as follows: [30] Grade I, mild retinal artery stenosis or sclerosis; Grade II, moderate arterial stenosis with arteriovenous crossing; Grade III, arterial stenosis and large arteriovenous crossover changes accompanied by bleeding, exudates, and cotton spots; and Grade IV, severe grade III with papilledema. Subject exclusion criteria were as follows: 1) history of eye surgery, diabetes, and/or nervous system disease; 2) unable to undergo MRI scanning for subjective and objective reasons; 3) alcohol intake >30 g/day; and 4) old or multiple cerebral infarctions in the MRI scan. We also recruited 31 healthy volunteers (16 males and 15 females) from various communities in Nanchang City, Jiangxi Province, China as the healthy control (HC) group, which was matched in terms of age and sex ratio to the HR group. All HCs were accorded with the following conditions: 1) no ophthalmic or neurologic diseases; 2) head MRI scan showing normal brain parenchyma; and 3) no contraindications for MRI.

### Correlation analysis

All participants were evaluated with the best corrected visual acuity (BCVA) test and Hospital Anxiety and Depression Scale (HADS). We used Prism 8 software (GraphPad Inc, La Jolla, CA, USA) to analyze the linear correlations between DC values of bilateral precuneus (BP) and BCVA of bilateral eyes; of the left cerebellum posterior lobe (LCPL) and depression score (DS); and of the left medial occipital gyrus (LMOG) and anxiety score (AS).

### Brain MRI and data processing

We have used an 8-channel head coil 3-T MR scanner (Trio; Siemens, Munich, Germany) to obtain T1-weighted images. MRIcro (https://www.MRIcro.com) was used to prefilter all functional data and DPARSF (https://www.rfmri.org/DPARSF) was used for preprocessing, with the first 10 volumes discarded to eliminate potential artifacts from scanner instability or the surrounding environment. We ultimately obtained 230 rolls of functional images covering the entire brain. Additional details can be found in a previous report [22].

### DC analysis

REST software (http://www.restfmri.net) was used for DC analysis. By filtering the preprocessed image (0.01 Hz<f<0.08 Hz), the noise which affected the image analysis and processing procedure can be eliminated. The DC values of HR patients and HCs were calculated. In addition, the following Fisher transformation formula was used to convert the correlation coefficient into a Z value, thus further improving the normality:

$$Z_{i}=\frac{DC_{i}-mean_{all}}{std_{all}}$$

where *Z_i_* is the Z score of the i^th^ voxel; *DC_i_* is the DC value of the i^th^ voxel; *mean_all_* is the average of all voxels in the brain structure; and *std_all_* is the standard deviation.

### Statistical analysis

Demographic and clinical data were analyzed using SPSS v20.0 software (SPSS Inc, Chicago, IL, USA). The relevant data of the subjects were tested for normality. To compare the differences between the HR group and the HC group, the independent sample t-test was used and the significance level was set as P<0.05. The 2-sample *t* test was used to detect the differences of mean DC values in each cerebral region between the HR and HC groups using same software; after Gaussian correction, the statistical threshold was also set to P<0.05. We identified specific cerebral regions with significant differences in DC values between the two group participants and inferred that differences in DC values in these cerebral regions could serve as potential markers for the differential diagnosis of HR. Receiver operating characteristic (ROC) curve analysis was used to test this hypothesis. Area under the ROC curve (AUC) values of 0.5–0.7 and 0.7–0.9 were considered to reflect low and high diagnostic accuracy, respectively.

## RESULTS

### Characteristics of the study population

The clinical course of HR in patients was 1.66±12.65 years. The HR and HC groups showed statistically significant differences in BCVA, systolic blood pressure, and diastolic blood pressure (P<0.05). However, there were no significant differences in sex (P>0.99), age (P=0.756), body weight (P=0.804), dominant hand (P>0.99), or heart rate (P=0.067). In addition, eight men and seven women in the HR group had a family history of hypertension, while none of the subjects in the HC group had a family history of hypertension. In terms of smoking status, there were 8 males and 7 females in both the HR group and HC group with smoking status (Table 2).

**Table 2:** Conditions of participants included in the study.

**Condition**	**HR**	**HCs**	**t**	P-value*
Male/female(M/F)	16/15	16/15	N/A	>0.99
Family history of hypertension(M/F)	8/7	N/A	N/A	N/A
Smoking status(M/F)	8/7	8/7	N/A	N/A
Age (years)	54.35±6.87	51.36±6.86	0.178	0.756
Weight (kg)	69.64±4.42	65.57±5.75	0.202	0.804
Handedness	31R	31R	N/A	>0.99
Duration of HR (years)	31.66±12.65	N/A	N/A	N/A
Best-corrected VA-left eye	0.66±0.17	1.05±0.25	-3.764	0.007
Best-corrected VA-right eye	0.57±0.21	1.10±0.20	-3.835	0.003
Confrontation VF	Full	Full	-N/A	N/A
SBP(mmHg)	166±25	116±18	9.037	<0.001
DBP(mmHg)	101±15	76±11	2.142	0.016
HR1(beats per minute)	69±12	62±14	0.825	0.067

* *p* <0.05 Independent t-tests comparing two groups, Data shown as mean ± standard deviation.

Abbreviations: DBP, diastolic blood pressure; HR, Hypertensive retinopathy; HR1, heart rate; HCs, normal controls; VA, visual acuity; N/A, not applicable; SBP, systolic blood pressure; VF, Visual field.

### Alterations in DC in HR

**Figure 2:**
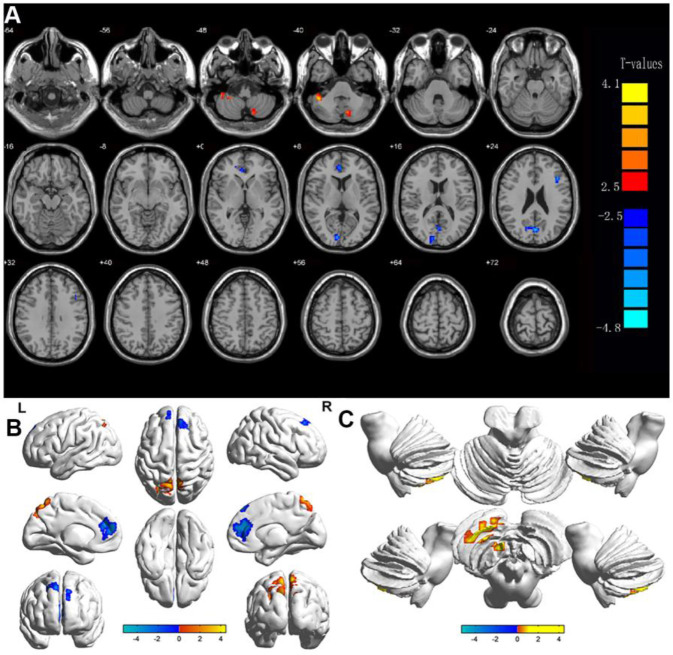
**Display of DC values in different brain regions.** Notes: Significant differences in DC were observed in (**A**, **B**) shows the changes in DC in the cerebral cortex, and (**C**) shows the changes in DC in the cerebellum. The yellow regions indicate higher DC values.

**Figure 3:**
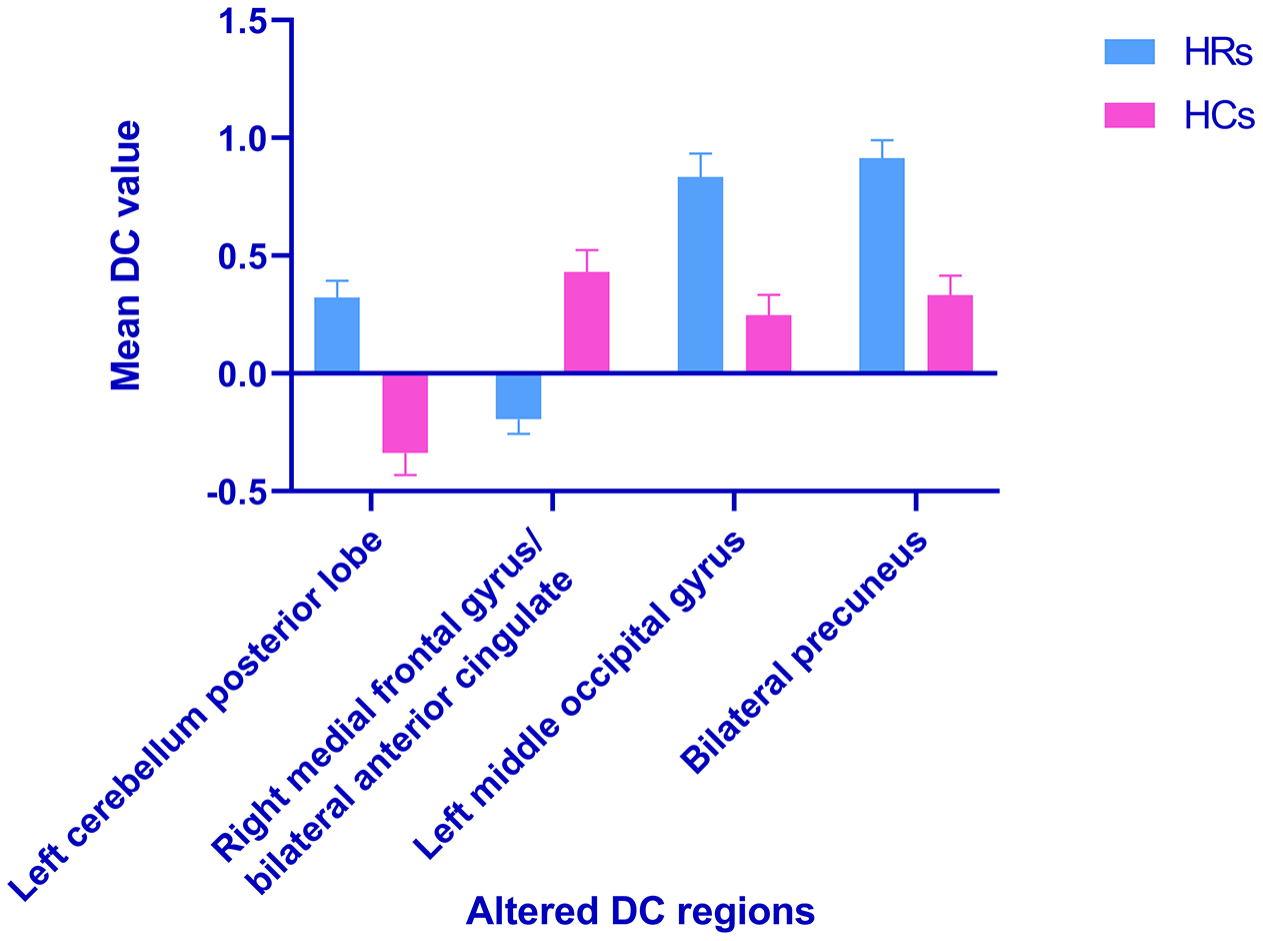
**Voxel-wise comparison of DC in the HR and healthy control group.** Notes: The mean DC values between the HR and HC groups. Abbreviations: DC, degree centrality; HRs, hypertensive retinopathy; HCs, healthy controls.

The DC values of right medial frontal gyrus (RMFG)/bilateral anterior cingulate (BAC) were significantly reduced, whereas the values of LCPL, LMOG, and BP were increased in HR patients compared to HCs (Figure 2). And the Figure 3 showed the differences in DC values between the 2 groups. DC values of RMFG/BAC, LCPL, LMOG, and BP differed significantly between the 2 groups (Table 3).

**Table 3:** Brain regions with significant difference in DC between HR patients and HCs.

**Conditions**	**Brain regions**	**BA**	**MNI coordinates**			**Peak voxels**	**t-value**
			**X**	**Y**	**Z**		
HRs>HCs	Left Cerebellum Posterior Lobe	/	-30	-66	-54	167	4.48
	Left Medial Occipital Gyrus	19	-30	-69	36	40	4.33
	Bilateral Precuneus	/	9	-60	66	234	3.98
HRs<HCs	Right Medial Frontal Gyrus/Bilateral Anterior Cingulate	9/32	12	45	21	557	-5.35

Notes: The statistical threshold was set at voxel with P<0.01 for multiple comparisons using false discovery rate. Abbreviations: DC, degree centrality; BA, Brodmann area; HR, hypertensive retinopathy; HC, healthy control; MNI, Montreal Neurological Institute.

### Linear correlation analysis

**Figure 4:**
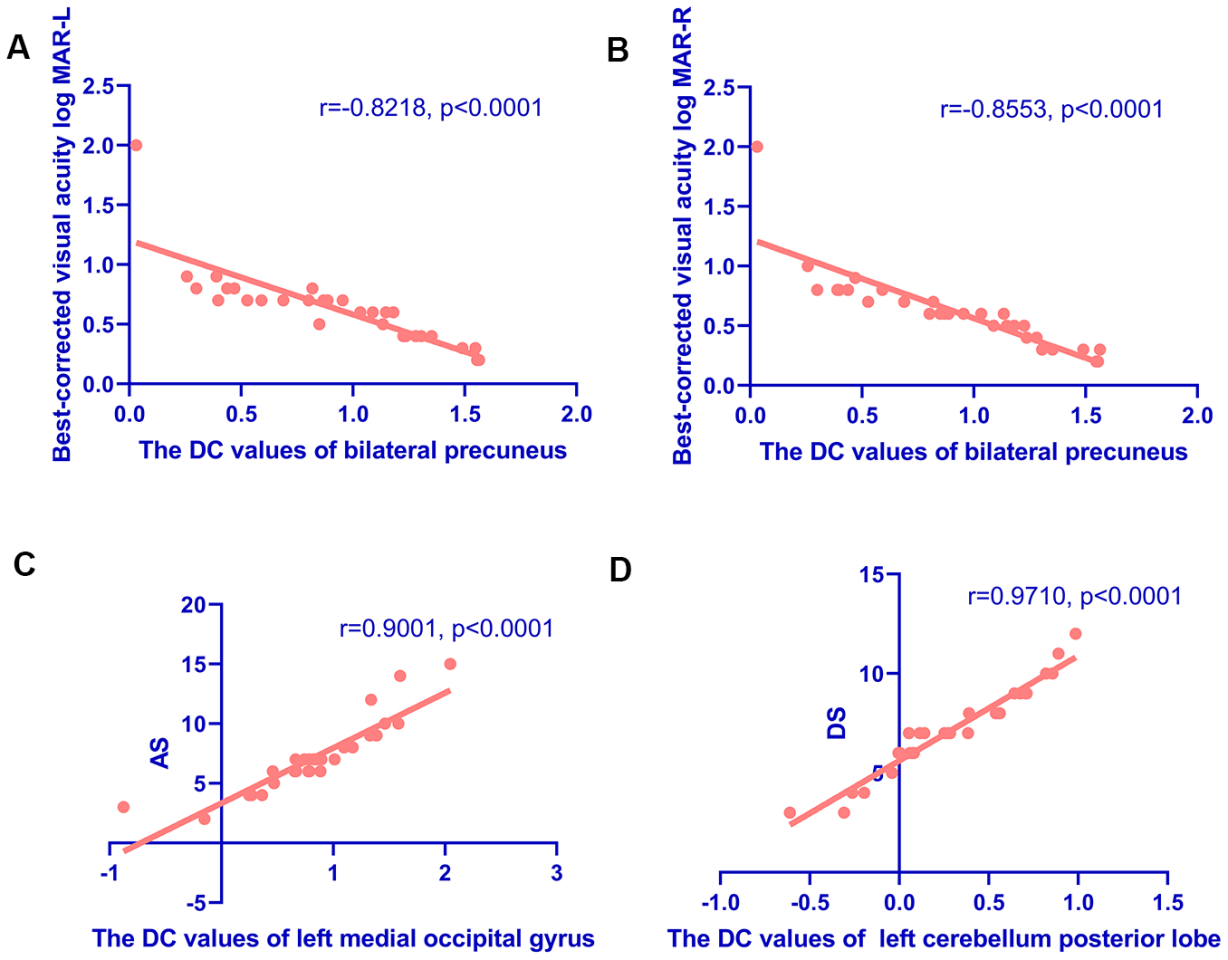
**Correlations between the DC values of different regions and the clinical behaviors in HR group.** (**A**, **B**) Correlations between the DC values of bilateral precuneus and best-corrected visual acuity. The DC values of bilateral precuneus were positively correlated with the values of BCVA-L(LogMAR) (r=-0.8218, p<0.0001) (**A**) and BCVA-R (LogMAR) (r=-0.8553, p<0.0001) (**B**). (**C**, **D**) Correlations between the DC values of specific cerebral regions and the Hospital Anxiety and Depression Scale. The DC values of the left middle occipital gyrus were positively correlated with AS (r=0.9001, p<0.0001) (**C**); and the DC values of the left cerebellum posterior lobe were positively correlated with the DS (r=0.9710, p<0.0001) (**D**). Abbreviations: DC, degree centrality; AS, anxiety scores; DS, depression scores.

The results showed that the DC values of BP were negatively correlated with the values of BCVA-L (LogMAR) (r=−0.8218, P<0.0001) (Figure 4A) and BCVA-R (LogMAR) (r=−0.8553, P<0.0001) (Figure 4B). DC values of LMOG and LCPL were positively correlated with AS (r=0.9001, P<0.0001) (Figure 4C) and DS (r=0.9710, P<0.0001) (Figure 4D), respectively.

### Diagnostic utility of altered DC values for HR

**Figure 5:**
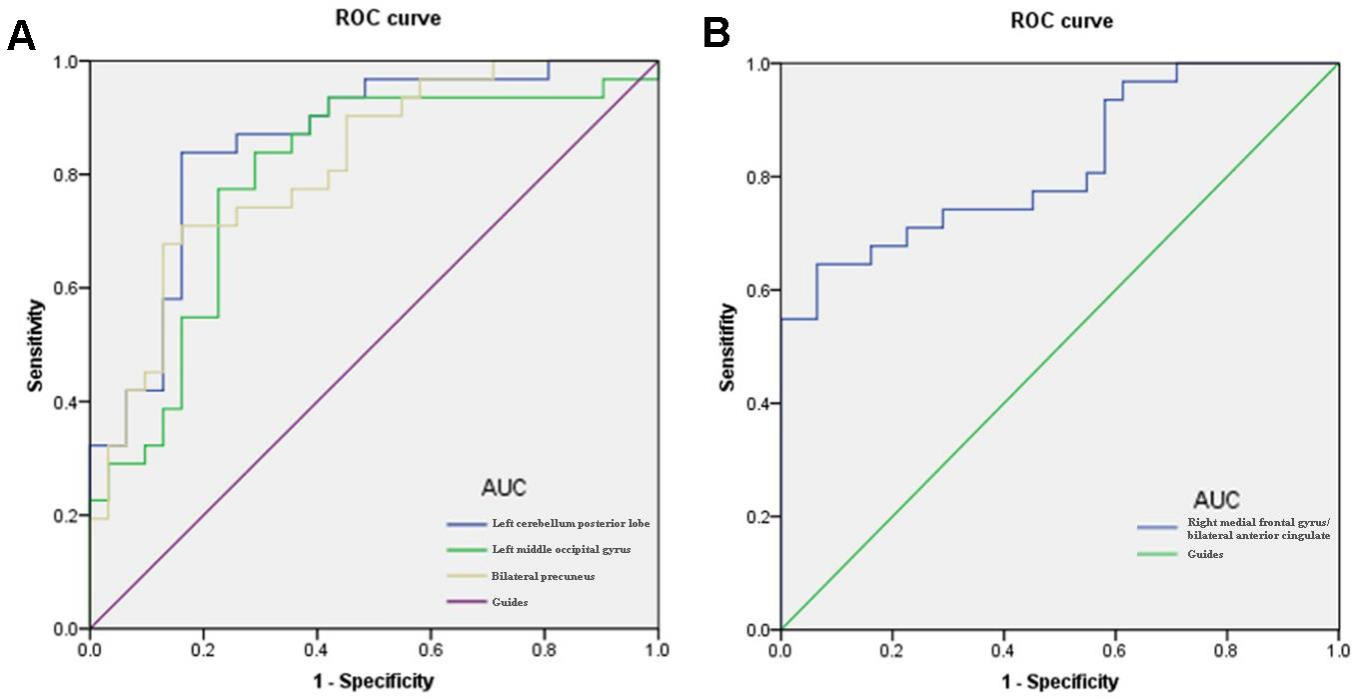
**ROC curve analysis of the mean DC values for altered brain regions.** Notes: (**A**) The area under the ROC curve were 0.855, (p<0.001; 95% CI: 0.760-0.951) for LCPL, LMOG 0.792 (p<0.001; 95% CI: 0.675-0.909), BP 0.816 (p<0.001; 95% CI: 0.711-0.920). (**B**) The area under the ROC curve was 0.822 (p<0.001; 95% CI: 0. 717-0.926) for RMFG/BAC. Abbreviations: DC, degree centrality; ROC, receiver operating characteristic; LCPL, left cerebellum posterior lobe; LMOG, left middle occipital gyrus; BP, bilateral precuneus; RMFG/BAC, right medial frontal gyrus/ bilateral anterior cingulate.

The DC values of the posterior cerebellum differed significantly between the HR patients and HCs. We speculated that this could serve as a diagnostic marker to distinguish the 2 groups. Further, we evaluated the mean DC values of specific brain regions in patients with HR by ROC curve analysis. The AUCs were as follows: LCPL, 0.855 (P<0.001; 95% confidence interval [CI]: 0.760–0.951); LMOG, 0.792 (P<0.001; 95% CI: 0.675–0.909); BP, 0.816 (P<0.001; 95% CI: 0.711–0.920); and RMFG/BAC, 0.822 (P<0.001; 95% CI: 0. 717–0.926) (Figure 5).

## DISCUSSION

**Figure 6:**
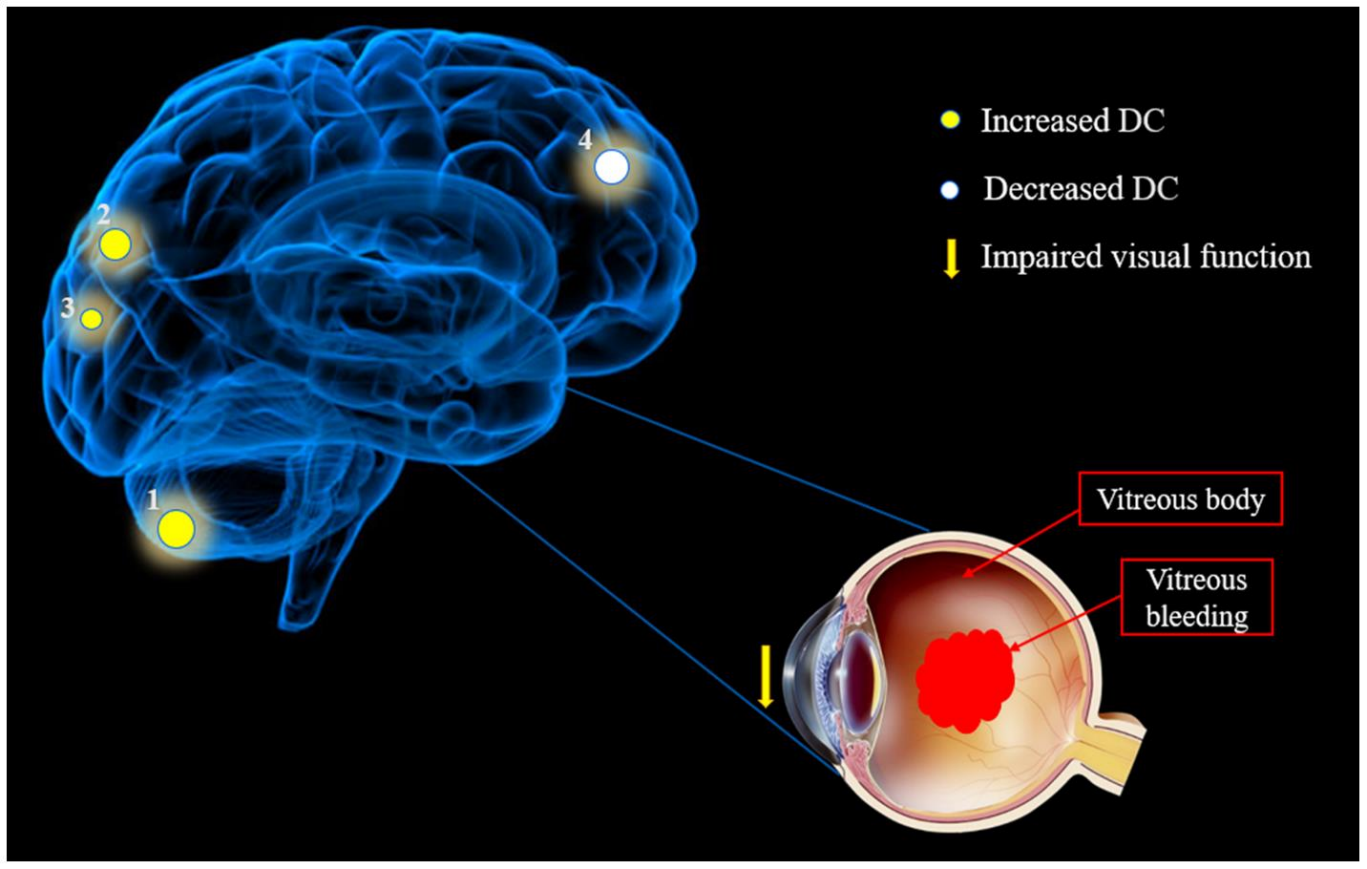
**The mean DC values of altered brain regions in the hypertensive retinopathy group.** Notes: Compared with the HCs, the DC values of the following regions were increased to various extents: 1- Cerebellum Posterior Lobe. L (-, t=4.4835), 2- Medial Occipital Gyrus. L (BA19, t=4.3309) and 3- Precuneus. B (-, t=3.9827), whereas the DC value of the following region was decreased: 4- Medial Frontal Gyrus/B Anterior Cingulate. R (-, t = -4.2564). The sizes of the spots denote the degree of quantitative changes. Abbreviations: DC, degree centrality; HCs, healthy controls; L, left; R, right; B, Bilateral; BA, Brodmann's area.

fMRI based on blood oxygenation levels is a useful method for investigating functional networks of the human brain. DC reflects the average strength of connectivity between each voxel and all voxels in the whole cerebrum, characterizing the significance of nodes in the network. DC is the most reliable centrality measure in large-scale network studies, which can quantify the importance of cerebral network nodes and reflect the attributes of functional network hubs [31]. In addition, DC method can provide objective and comprehensive information of resting-state functional connectivity in the whole brain network, and this method has more advantages than functional connectivity and regional homogeneity [32]. Finally, DC has the characteristics of noninvasive and convenient, which makes it widely used in the study of cerebral function. Therefore, we applied rs-fMRI and DC analysis to determine whether changes in FC in the brain have diagnostic utility for HR. Rs-fMRI results showed that compared with HCs, LCPL, LMOG and BP DC values of HR patients increased, but RMFG/BAC has a reduced DC value (Figure 6).

The posterior lobe is located between the cerebellar and posterolateral fissures, occupying most of the cerebellum. In addition to participating in coordinated motion control, the posterior cerebellar lobe is involved in cognitive, emotional, and visual processing. Lesions in this area can result in cerebellar cognitive affective syndrome, [33] which is characterized by impaired visuospatial processing, language skills, and emotional regulation. The cerebellum also regulates mood and cognitive experience, [34] and cerebellar dysfunction has been linked to mood disorders including bipolar disorder, and depression [35]. In our research, the intrinsic cerebral FC of LCPL was enhanced in HR patients. We used the HADS to assess anxiety and depression in all participants and found that the DC value of LCPL was positively correlated with DS in the HR group. This result reflected that the enhancement of cerebral FC in LCPL of HR patients was closely related to depression. We considered the negative impact of diminution of vision due to HR on patients' quality of life and emotional intelligence. This effect was reflected in the significant enhancement of cerebral FC in LCPL. In addition, alternations in the neural activity of LCPL may also be the underlying pathological basis of depression in HR patients. Therefore, in the process of clinical diagnosis, if the LCPL functional activities of HR patients were found to be abnormal, it was necessary to closely observe their emotional activities to avoid the accumulation of negative emotions and affect their physical health.

The occipital lobe contains most of the visual cortex and functions as a hub connecting visual input to higher-order cognitive processing centers. The MOG is located outside the striatum of the visual cortex and is engaged in visual motor processing and spatial analysis [36]. Changes in local nerve activity in the occipital lobe are linked to the speed of visual information processing, spatial memory, and overall cognitive ability. The thickness of the MOG is significantly increased in patients with early-onset blindness, [37] which is considered to be the result of compensation. Of the multiple brain regions related to depression and anxiety, [38] the OG is associated with personal emotions [39]. An rs-fMRI analysis of alterations in baseline cerebral activity of adult patients with social anxiety disorder who had not received pharmacologic treatment found that the amplitude of low-frequency fluctuation of bilateral MOG was significantly increased, indicating changes in brain function [40]. Thus, aberrant activities in the MOG were related to negative emotions such as anxiety. In our study, the DC value of LMOG was increased in HR patients and was positively correlated with AS. As indicated by the above analysis, the main functions of MOG involved the processing of visual information and emotional intelligence. We speculated that the decreased visual acuity caused by HR, and the MOG region significantly enhanced the functional activities of this cerebral region to compensate for the abnormal visual acuity, and accelerated the processing of visual information. On the other hand, due to the limitation of the compensatory effect, the vision of HR patients was still difficult to return to normal, and the patients may have abnormal emotional states such as anxiety. Our study found that the functional activity of LMOG was closely related to anxiety. This further validated our hypothesis. Therefore, clinical examination should pay close attention to the potential relationship between functional activity in this cerebral region and negative emotion and the functional state of the visual system.

The precuneus is part of the posterior parietal cortex, located on the medial hemisphere of the cerebrum. Some scholars pointed out that individuals with early-onset blindness showed activation of the precuneus in Brodmann’s area 7 during visuospatial imaging tasks [41]. Additionally, the precuneus plays an important role in guiding spatial attention, attentional shifts, plot memory and retrieval, and encoding and extracting spatial positions [42]. It also coordinates the neural networks of the temporal, parietal, and occipital lobes to extract detailed visuospatial information [43]. In this study, the DC value of the BP was increased in HR patients and was negatively correlated with the BCVA (LogMAR) of bilateral eyes. In our analysis above, it was shown that the main role of precuneus was to participate in the analysis and processing of spatial information, which was closely related to the visual system. Similar to LMOG, BP cerebral regions were also involved in the compensatory restoration of vision in HR patients. In addition, the linear regression analysis showed that the worse the visual acuity of patients caused by HR, the more significant the compensation effect of BP was, and the more active the functional activity of this cerebral region was, and the higher the corresponding DC value was. The DC value of LMOG was contrastively analyzed, and no correlation was found between LMOG and BCVA. Therefore, we infer that BP was the most active region in the specific cerebral regions involved in compensating visual information in HR patients. The important role of precuneus in the formation of vision was further verified.

The MFG occupies a large portion of the frontal lobe between the superior frontal and subfrontal sulci. The frontal lobe is involved in visual positioning, processing of spatial information, and transmission of visual information to the oculomotor nerve complex [44]. The MFG is an important part of the parietal prefrontal pathway and participates in visuospatial working memory [45]. Our research indicated that the DC value of the RMFG in the HR group was lower than in HCs, suggesting damage to the frontal lobe that may be related to the reduced vision in HR patients. The anterior cingulate gyrus (ACG) is located on the inner side of the frontal lobe, participating in the regulation of emotional learning and emotional evaluation of internal or external stimuli. The anterior and lower regions are deemed to be engaged in emotional regulation and contribute to the processing of anxiety, fear, sadness, and other emotions [46]. Clinical studies have shown that removal of the ACG and surrounding cortical tissue alleviated anxiety, depression, and other negative emotions in patients with intractable pain [47]. However, in our study, no correlation was found between RMFG/BAC and negative emotions in HR patients. Compared with HC group, the functional activity of this cerebral region was significantly decreased in HR group. The above studies indicated that the RMFG/BAC cerebral region was involved in the transmission of visual information. We hypothesized that it was precisely because the visual information acquired by vision loss was reduced that the required transmission activity was also reduced. Therefore, the FC of this cerebral region was also significantly reduced, and the corresponding DC value was also lower than that of the normal group. We further inferred that the functional activity of this cerebral region changed in the same direction as the visual acuity. This result had important significance in clinical examination.

Based on our findings above, we constructed a table of the functions corresponding with various brain region, and the effects of alterations associated with HR on functional activities (Table 4).

**Table 4:** Changes in specific brain regions and their potential functional effects.

**Brain regions**	**Experimental result**	**Brain function**
left cerebellum posterior lobe	HRs>HCs	Motor control, cognitive, emotional and visual processing
left middle occipital gyrus	HRs>HCs	Visual motor processing, spatial analysis, depression and anxiety
bilateral precuneus	HRs>HCs	Spatial attention, memory and visual spatial information processing
right medial frontal gyrus /bilateral anterior cingulate	HRs<HCs	Visual information transmission, visual spatial working memory and emotion regulation

Abbreviation: HR, hypertensive retinopathy; HC, healthy control.

In summary, this study found abnormal alteration in the FC of specific cerebral regions in HR patients. These results can lay a foundation for further study of the pathophysiological mechanism of HR. And provide data support for the vision health protection of middle-aged and elderly people.

### Ethical statement

All research methods were approved by the committee of the medical ethics of the First Affiliated Hospital of Nanchang University and were in accordance with the 1964 Helsinki declaration and its later amendments or comparable ethical standards. All subjects were explained the purpose, method, potential risks and signed an informed consent form.

## References

[1] Fraser-Bell S, Symes R, Vaze A. Hypertensive eye disease: a review. Clin Exp Ophthalmol. 2017; 45:45–53. 10.1111/ceo.12905 27990740

[2] Klein R, Klein BE, Moss SE. The relation of systemic hypertension to changes in the retinal vasculature: the Beaver Dam Eye Study. Trans Am Ophthalmol Soc. 1997; 95:329–48. 9440178 PMC1298366

[3] Konstantinidis L, Guex-Crosier Y. Hypertension and the eye. Curr Opin Ophthalmol. 2016; 27:514–21. 10.1097/ICU.0000000000000307 27662019

[4] Bhargava M, Ikram MK, Wong TY. How does hypertension affect your eyes? J Hum Hypertens. 2012; 26:71–83. 10.1038/jhh.2011.37 21509040

[5] Modi P, Arsiwalla T. Hypertensive Retinopathy. In: StatPearls. Treasure Island (FL): StatPearls Publishing. 2020. https://www.ncbi.nlm.nih.gov/books/NBK525980/30252236

[6] Harjasouliha A, Raiji V, Garcia Gonzalez JM. Review of hypertensive retinopathy. Dis Mon. 2017; 63:63–69. 10.1016/j.disamonth.2016.10.002 27931746

[7] Patton N, Aslam TM, MacGillivray T, Deary IJ, Dhillon B, Eikelboom RH, Yogesan K, Constable IJ. Retinal image analysis: concepts, applications and potential. Prog Retin Eye Res. 2006; 25:99–127. 10.1016/j.preteyeres.2005.07.001 16154379

[8] van der Veen PH, Geerlings MI, Visseren FL, Nathoe HM, Mali WP, van der Graaf Y, Muller M, and SMART Study Group. Hypertensive Target Organ Damage and Longitudinal Changes in Brain Structure and Function: The Second Manifestations of Arterial Disease-Magnetic Resonance Study. Hypertension. 2015; 66:1152–58. 10.1161/HYPERTENSIONAHA.115.06268 26503971

[9] Wong TY, Klein R, Klein BE, Tielsch JM, Hubbard L, Nieto FJ. Retinal microvascular abnormalities and their relationship with hypertension, cardiovascular disease, and mortality. Surv Ophthalmol. 2001; 46:59–80. 10.1016/s0039-6257(01)00234-x 11525792

[10] Ong YT, Wong TY, Klein R, Klein BE, Mitchell P, Sharrett AR, Couper DJ, Ikram MK. Hypertensive retinopathy and risk of stroke. Hypertension. 2013; 62:706–11. 10.1161/HYPERTENSIONAHA.113.01414 23940194 PMC4085393

[11] Hanff TC, Sharrett AR, Mosley TH, Shibata D, Knopman DS, Klein R, Klein BE, Gottesman RF. Retinal microvascular abnormalities predict progression of brain microvascular disease: an atherosclerosis risk in communities magnetic resonance imaging study. Stroke. 2014; 45:1012–17. 10.1161/STROKEAHA.113.004166 24549866 PMC4191897

[12] Cooper LS, Wong TY, Klein R, Sharrett AR, Bryan RN, Hubbard LD, Couper DJ, Heiss G, Sorlie PD. Retinal microvascular abnormalities and MRI-defined subclinical cerebral infarction: the Atherosclerosis Risk in Communities Study. Stroke. 2006; 37:82–86. 10.1161/01.STR.0000195134.04355.e5 16306463

[13] Wong TY, Mosley TH Jr, Klein R, Klein BE, Sharrett AR, Couper DJ, Hubbard LD, and Atherosclerosis Risk in Communities Study. Retinal microvascular changes and MRI signs of cerebral atrophy in healthy, middle-aged people. Neurology. 2003; 61:806–11. 10.1212/01.wnl.0000086372.05488.8d 14504325

[14] Yatsuya H, Folsom AR, Wong TY, Klein R, Klein BE, Sharrett AR, and ARIC Study Investigators. Retinal microvascular abnormalities and risk of lacunar stroke: Atherosclerosis Risk in Communities Study. Stroke. 2010; 41:1349–55. 10.1161/STROKEAHA.110.580837 20522816 PMC2894269

[15] Biswal BB, Mennes M, Zuo XN, Gohel S, Kelly C, Smith SM, Beckmann CF, Adelstein JS, Buckner RL, Colcombe S, Dogonowski AM, Ernst M, Fair D, et al. Toward discovery science of human brain function. Proc Natl Acad Sci USA. 2010; 107:4734–39. 10.1073/pnas.0911855107 20176931 PMC2842060

[16] Lu H, Zuo Y, Gu H, Waltz JA, Zhan W, Scholl CA, Rea W, Yang Y, Stein EA. Synchronized delta oscillations correlate with the resting-state functional MRI signal. Proc Natl Acad Sci USA. 2007; 104:18265–69. 10.1073/pnas.0705791104 17991778 PMC2084331

[17] Matsuoka T, Imai A, Fujimoto H, Kato Y, Shibata K, Nakamura K, Yokota H, Yamada K, Narumoto J. Reduced Pineal Volume in Alzheimer Disease: A Retrospective Cross-sectional MR Imaging Study. Radiology. 2018; 286:239–48. 10.1148/radiol.2017170188 28745939

[18] Lowe MJ, Phillips MD, Lurito JT, Mattson D, Dzemidzic M, Mathews VP. Multiple sclerosis: low-frequency temporal blood oxygen level-dependent fluctuations indicate reduced functional connectivity initial results. Radiology. 2002; 224:184–92. 10.1148/radiol.2241011005 12091681

[19] Hernández-Díaz ZM, Peña-Sánchez M, González-Quevedo Monteagudo A, González-García S, Arias-Cadena PA, Brown-Martínez M, Betancourt-Loza M, Cordero-Eiriz A. Cerebral Small Vessel Disease Associated with Subclinical Vascular Damage Indicators in Asymptomatic Hypertensive Patients. Behav Sci (Basel). 2019; 9:91. 10.3390/bs9090091 31443428 PMC6769830

[20] Beauchet O, Celle S, Roche F, Bartha R, Montero-Odasso M, Allali G, Annweiler C. Blood pressure levels and brain volume reduction: a systematic review and meta-analysis. J Hypertens. 2013; 31:1502–16. 10.1097/HJH.0b013e32836184b5 23811995

[21] Zuo XN, Ehmke R, Mennes M, Imperati D, Castellanos FX, Sporns O, Milham MP. Network centrality in the human functional connectome. Cereb Cortex. 2012; 22:1862–75. 10.1093/cercor/bhr269 21968567

[22] Wang Y, Jiang L, Wang XY, Chen W, Shao Y, Chen QK, Lv JL. Evidence of altered brain network centrality in patients with diabetic nephropathy and retinopathy: an fMRI study using a voxel-wise degree centrality approach. Ther Adv Endocrinol Metab. 2019; 10:2042018819865723. 10.1177/2042018819865723 31384421 PMC6661786

[23] Cai F, Gao L, Gong H, Jiang F, Pei C, Zhang X, Zeng X, Huang R. Network Centrality of Resting-State fMRI in Primary Angle-Closure Glaucoma Before and After Surgery. PLoS One. 2015; 10:e0141389. 10.1371/journal.pone.0141389 26506229 PMC4624709

[24] Huang X, Li HJ, Peng DC, Ye L, Yang QC, Zhong YL, Zhou FQ, Shao Y. Altered brain network centrality in patients with late monocular blindness: a resting-state fMRI study. Arch Med Sci. 2019; 15:1301–07. 10.5114/aoms.2019.87133 31572477 PMC6764322

[25] Tan G, Dan ZR, Zhang Y, Huang X, Zhong YL, Ye LH, Rong R, Ye L, Zhou Q, Shao Y. Altered brain network centrality in patients with adult comitant exotropia strabismus: A resting-state fMRI study. J Int Med Res. 2018; 46:392–402. 10.1177/0300060517715340 28679330 PMC6011327

[26] Wang H, Chen T, Ye L, Yang QC, Wei R, Zhang Y, Jiang N, Shao Y. Network centrality in patients with acute unilateral open globe injury: A voxel-wise degree centrality study. Mol Med Rep. 2017; 16:8295–300. 10.3892/mmr.2017.7635 28983610

[27] Hu YX, He JR, Yang B, Huang X, Li YP, Zhou FQ, Xu XX, Zhong YL, Wang J, Wu XR. Abnormal resting-state functional network centrality in patients with high myopia: evidence from a voxel-wise degree centrality analysis. Int J Ophthalmol. 2018; 11:1814–20. 10.18240/ijo.2018.11.13 30450313 PMC6232325

[28] Banerjee A, Nayak B, Verma G, Parija S. Resolution of grade IV hypertensive retinopathy in an adult with pheochromocytoma: post-tumor resection. BMJ Case Rep. 2020; 13:e231245. 10.1136/bcr-2019-231245 32060107 PMC7046403

[29] Whelton PK, Carey RM, Aronow WS, Casey DE Jr, Collins KJ, Dennison Himmelfarb C, DePalma SM, Gidding S, Jamerson KA, Jones DW, MacLaughlin EJ, Muntner P, Ovbiagele B, et al. 2017 ACC/AHA/AAPA/ABC/ACPM/AGS/APhA/ASH/ASPC/NMA/PCNA Guideline for the Prevention, Detection, Evaluation, and Management of High Blood Pressure in Adults: A Report of the American College of Cardiology/American Heart Association Task Force on Clinical Practice Guidelines. Hypertension. 2018; 71:e13–115. 10.1161/HYP.0000000000000065 29133356

[30] Keith NM, Wagener HP, Barker NW. Some different types of essential hypertension: their course and prognosis. Am J Med Sci. 1974; 268:336–45. 10.1097/00000441-197412000-00004 4616627

[31] Wu K, Liu M, He L, Tan Y. Abnormal degree centrality in delayed encephalopathy after carbon monoxide poisoning: a resting-state fMRI study. Neuroradiology. 2020; 62:609–16. 10.1007/s00234-020-02369-0 31955235 PMC7186243

[32] Chen P, Hu R, Gao L, Wu B, Peng M, Jiang Q, Wu X, Xu H. Abnormal degree centrality in end-stage renal disease (ESRD) patients with cognitive impairment: a resting-state functional MRI study. Brain Imaging Behav. 2020. [Epub ahead of print]. 10.1007/s11682-020-00317-3 32902798

[33] Schmahmann JD. The cerebellum and cognition. Neurosci Lett. 2019; 688:62–75. 10.1016/j.neulet.2018.07.005 29997061

[34] Borsook D, Moulton EA, Tully S, Schmahmann JD, Becerra L. Human cerebellar responses to brush and heat stimuli in healthy and neuropathic pain subjects. Cerebellum. 2008; 7:252–72. 10.1007/s12311-008-0011-6 18418691

[35] Bledsoe JC, Semrud-Clikeman M, Pliszka SR. Neuroanatomical and neuropsychological correlates of the cerebellum in children with attention-deficit/hyperactivity disorder--combined type. J Am Acad Child Adolesc Psychiatry. 2011; 50:593–601. 10.1016/j.jaac.2011.02.014 21621143 PMC3104210

[36] Fortin A, Ptito A, Faubert J, Ptito M. Cortical areas mediating stereopsis in the human brain: a PET study. Neuroreport. 2002; 13:895–98. 10.1097/00001756-200205070-00032 11997709

[37] Anurova I, Renier LA, De Volder AG, Carlson S, Rauschecker JP. Relationship Between Cortical Thickness and Functional Activation in the Early Blind. Cereb Cortex. 2015; 25:2035–48. 10.1093/cercor/bhu009 24518755 PMC4494021

[38] Park BY, Park H. Connectivity differences between adult male and female patients with attention deficit hyperactivity disorder according to resting-state functional MRI. Neural Regen Res. 2016; 11:119–25. 10.4103/1673-5374.175056 26981099 PMC4774203

[39] Pannekoek JN, van der Werff SJ, van Tol MJ, Veltman DJ, Aleman A, Zitman FG, Rombouts SA, van der Wee NJ. Investigating distinct and common abnormalities of resting-state functional connectivity in depression, anxiety, and their comorbid states. Eur Neuropsychopharmacol. 2015; 25:1933–42. 10.1016/j.euroneuro.2015.08.002 26321187

[40] Qiu C, Feng Y, Meng Y, Liao W, Huang X, Lui S, Zhu C, Chen H, Gong Q, Zhang W. Analysis of Altered Baseline Brain Activity in Drug-Naive Adult Patients with Social Anxiety Disorder Using Resting-State Functional MRI. Psychiatry Investig. 2015; 12:372–80. 10.4306/pi.2015.12.3.372 26207132 PMC4504921

[41] Vanlierde A, De Volder AG, Wanet-Defalque MC, Veraart C. Occipito-parietal cortex activation during visuo-spatial imagery in early blind humans. Neuroimage. 2003; 19:698–709. 10.1016/s1053-8119(03)00153-8 12880800

[42] Cavanna AE, Trimble MR. The precuneus: a review of its functional anatomy and behavioural correlates. Brain. 2006; 129:564–83. 10.1093/brain/awl004 16399806

[43] Schott BH, Wüstenberg T, Lücke E, Pohl IM, Richter A, Seidenbecher CI, Pollmann S, Kizilirmak JM, Richardson-Klavehn A. Gradual acquisition of visuospatial associative memory representations via the dorsal precuneus. Hum Brain Mapp. 2019; 40:1554–70. 10.1002/hbm.24467 30430687 PMC6865719

[44] Mazzarella E, Ramsey R, Conson M, Hamilton A. Brain systems for visual perspective taking and action perception. Soc Neurosci. 2013; 8:248–67. 10.1080/17470919.2012.761160 23350907

[45] Ren Z, Zhang Y, He H, Feng Q, Bi T, Qiu J. The Different Brain Mechanisms of Object and Spatial Working Memory: Voxel-Based Morphometry and Resting-State Functional Connectivity. Front Hum Neurosci. 2019; 13:248. 10.3389/fnhum.2019.00248 31379543 PMC6659551

[46] Bush G, Luu P, Posner MI. Cognitive and emotional influences in anterior cingulate cortex. Trends Cogn Sci. 2000; 4:215–22. 10.1016/s1364-6613(00)01483-2 10827444

[47] Yen CP, Kuan CY, Sheehan J, Kung SS, Wang CC, Liu CK, Kwan AL. Impact of bilateral anterior cingulotomy on neurocognitive function in patients with intractable pain. J Clin Neurosci. 2009; 16:214–19. 10.1016/j.jocn.2008.04.008 19101146

